# Sustained Immunoparalysis in Endotoxin-Tolerized Monocytic Cells

**DOI:** 10.1155/2020/8294342

**Published:** 2020-06-13

**Authors:** Christina K. Weisheit, Alexandra Klüners, Lennart Wild, Alexandra Casalter, Stefanie Heilmann-Heimbach, Sugirthan Sivalingam, Jan L. Kleiner, Stefan F. Ehrentraut, Andreas Hoeft, Stilla Frede, Heidi Ehrentraut

**Affiliations:** ^1^Department of Anesthesiology and Intensive Care Medicine, University Hospital Bonn, Germany; ^2^Department of Genomics, Life & Brain Center, Institute of Human Genetics, University of Bonn, Germany

## Abstract

Sepsis is associated with a strong inflammatory reaction triggering a complex and prolonged immune response. Septic patients have been shown to develop sustained immunosuppression due to a reduced responsiveness of leukocytes to pathogens. Changes in cellular metabolism of leukocytes have been linked to this phenomenon and contribute to the ongoing immunological derangement. However, the underlying mechanisms of these phenomena are incompletely understood. In cell culture models, we mimicked LPS tolerance conditions to provide evidence that epigenetic modifications account for monocyte metabolic changes which cause immune paralysis in restimulated septic monocytes. In detail, we observed differential methylation of CpG sites related to metabolic activity in human PBMCs 18 h after septic challenge. The examination of changes in immune function and metabolic pathways was performed in LPS-tolerized monocytic THP-1 cells. Passaged THP-1 cells, inheriting initial LPS challenge, presented with dysregulation of cytokine expression and oxygen consumption for up to 7 days after the initial LPS treatment. Proinflammatory cytokine concentrations of TNF*α* and IL1*β* were significantly suppressed following a second LPS challenge (*p* < 0.001) on day 7 after first LPS stimulation. However, the analysis of cellular metabolism did not reveal any noteworthy alterations between tolerant and nontolerant THP-1 monocytes. No quantitative differences in ATP and NADH synthesis or participating enzymes of energy metabolism occurred. Our data demonstrate that the function and epigenetic modifications of septic and tolerized monocytes can be examined in vitro with the help of our LPS model. Changes in CpG site methylation and monocyte function point to a correlation between epigenetic modification in metabolic pathways and reduced monocyte function under postseptic conditions.

## 1. Introduction

Sepsis is associated with an interplay of strong proinflammatory and anti-inflammatory responses. However, the latter may exacerbate host defense mechanisms and alter immune cell functions [1]. Prolonged immune dysfunction in patients coping with sepsis compromises their outcome upon secondary infections. These patients' leukocytes displayed immunoparalysis, characterized by decreased inflammation and a generalized metabolic defect on the level of glycolysis and oxidative metabolism. Depending on the dose and type of a first trigger, a second stimulation can induce diverse responses [2, 3]. Epigenetic memory may provoke tolerance or training, i.e., to reduce or enhance the immune reaction [4, 5]. Endotoxin tolerance is a crucial homeostatic mechanism that prevents the excessive stimulation of the innate immune response upon sustained or recurrent toll-like receptor (TLR) stimulation [6]. Endotoxin tolerance is considered a type of innate immune memory, a condition describing tolerance towards pathogens, characterized by innate immune hyporesponsiveness or immunoparalysis [7, 8].

Prestimulation of monocytes or macrophages with high doses of lipopolysaccharide (LPS) can induce LPS tolerance [9]. Low doses of LPS, however, can augment the response to the second LPS trigger, resulting in training instead of tolerance, indicating that effects on memory are dose dependent [10, 11].

During infection, bacterial molecular patterns such as LPS affect signal transduction on various levels. Modifications of the TLR 4 signaling cascade, altered pro- and anti-inflammatory cytokine and chemokine levels, changes in the activation of transcription factors, and epigenomic mechanisms may reprogram the host's response [2]. Epigenetic effects including DNA methylation can be of transient or sustained character [12]. Their influence on inflammatory and immunological processes is a matter of ongoing investigations. So far, little is known since the complexity of interactions on clinical, cellular, and molecular levels impedes transfer and reproduction in cellular models. DNA methylation is the most stable mark, followed by shorter lived histone methylations, both enabling a preservation of alterations after cell division [13].

The vast majority of studies examining the mechanisms of innate immunotolerance lacks long-term observations. Proliferation of cells is a prerequisite when investigating how parent cells pass on immunomodulatory effects, potentially via epigenetic processes [14]. However, proliferation of PBMCS under culturing conditions is difficult to achieve. Stimulation with selective growth factors alters the metabolic response and cellular effector function after stimulation. Therefore, we developed a long-term model of LPS-pretreated monocytic THP-1 cells, undergoing continuous passaging in LPS-free media, and analyzed their phenotype.

Monocytes arise from myeloid progenitor cells in the bone marrow. Responding to differentiation stimuli and growth factors, monocytes enter the bloodstream. Upon inflammatory signals, monocytes migrate to infected tissues and start differentiation. Monocytes are central to the development of both systemic inflammatory response syndrome and the compensatory anti-inflammatory response syndrome [15, 16]. Phagocytosis, antigen presentation, and cytokine production of monocytes are influenced by metabolic modulation [17]. LPS induces reprogramming in macrophages and dendritic cells via TLR4 by suppression of oxidative phosphorylation, increase in glucose uptake and glycolysis, and other metabolic pathways [18].

We intended to analyze how immunocompromisation is reflected on the level of CpG methylation. Furthermore, the progression of immune dysfunction and metabolic alteration over an extended period of time was our underlying question.

## 2. Materials and Methods

### 2.1. PBMC Isolation and LPS Stimulation

Fresh peripheral blood was drawn from four healthy volunteers. PBMC isolation was performed by overlaying 6 ml of blood over each 3 ml of Histopaque 1077 and 1119 (Sigma). 15 ml tubes were centrifuged at room temperature (RT) for 30 min at 700 × g. The resulting PBMC layer was retrieved and washed three times (10 min, 300 × g) with PBS at RT. Cells were resuspended at the indicated cell number in RPMI 1640 (Lonza, Basel, Switzerland) supplemented with 10% heat-inactivated fetal bovine serum (FBS, Applichem, Darmstadt, Germany).

**Figure 1:**
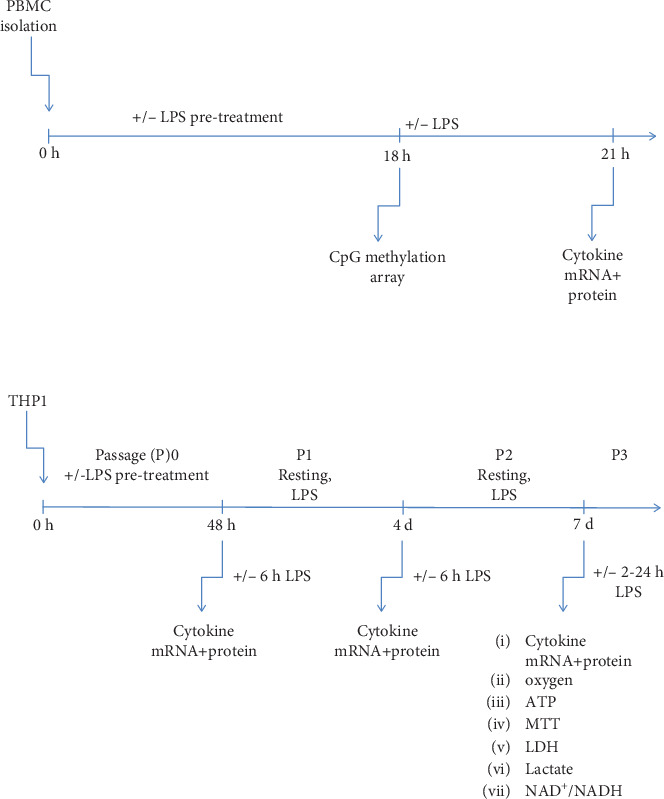
Study design. Timeline of PBMC and THP-1 cultivation, LPS treatment, and sampling.

PBMCs of each subject were incubated with 10 ng LPS/ml (*E. coli* LPS 0111:B4, Sigma, St. Louis, MO, USA) or medium control (Figure 1). After 18 hours, samples were exposed to a higher dose of LPS (100 ng/ml media) or medium control. Three hours later, samples were centrifuged for 5 min at 800 × g and 4°C. Supernatants and cell pellets were separated and kept at -80°C until further processing.

### 2.2. DNA Isolation, Bisulfite Conversion, and Infinium Methylation EPIC Bead Chip Array

PBMCs were harvested from four additional healthy volunteers. For each subject, 10^7^ PBMCs/ml were incubated with 10 ng LPS/ml or PBS for 18 hours and harvested by centrifugation. DNA was isolated using a Genomic DNA Isolation Kit (Machery Nagel, Düren, Germany). The isolated DNA was stored at -20°C. Bisulfite conversion of DNA was performed utilizing Qiagen EpiTect Kit (Qiagen, Hilden, Germany) according to the manufacturer's protocol. Infinium Methylation EPIC Bead Chip Array (Illumina, San Diego, USA) was run as published elsewhere [19].

### 2.3. THP-1 Culture and Passaging Experiment

THP-1 cells were obtained from ATCC (Manassas, VA, USA) and cultured in RPMI 1640 (Lonza) supplemented with 10% heat-inactivated FBS. For maintenance, THP-1 culture cells were split every 2 to 3 days and resuspended to 10^6^ cells per ml medium.

Prior to stimulation, cells from T125 flasks were pooled, counted, and resuspended at 10^6^ cells/ml media. Cells were seeded into T75 flasks and stimulated once with either LPS (1 *μ*g/ml medium) or PBS as control (passage (P)0) (Figure 1). Before passaging, cells were counted, centrifuged (400 × g for 5 min), and resuspended at 10^6^ cells/ml LPS-free fresh media each time. In each passaging cycle, a portion of cells from both treatment groups was transferred to a new dish and received a second stimulation with LPS (1 *μ*g/ml medium) or PBS.

### 2.4. RNA Isolation and cDNA Synthesis

RNA was isolated from 1 × 10^6^ THP-1 cells or 6 × 10^6^ PBMCs per sample. Cells were resuspended in 1 ml TRIzol (Thermo Fisher Scientific). After the addition of 200 *μ*l chloroform (Applichem, Darmstadt, Germany), suspension was repeatedly inverted, followed by 10 min incubation at room temperature. Phases were separated by centrifugation (12,000 × g) at 4°C for 15 min. The upper aqueous phase was transferred into a new RNase-free reaction tube. The RNA precipitated after addition of 500 *μ*l isopropanol and 10 min incubation at room temperature. After centrifugation (12,000 × g, 10 min, 4°C), the supernatant was discarded and the pellet was washed twice using 1 ml 75% ethanol in DEPC-H_2_O, followed by centrifugation (10,000 × g, 5 min, 4°C). The air-dried RNA pellet was resuspended in 20 *μ*l DEPC-H_2_O. RNA concentration was determined spectrophotometrically (NanoDrop, Thermo Fisher Scientific). High-capacity cDNA Reverse Transcription Kit (Thermo Fisher Scientific) was utilized to convert 2 *μ*g total RNA to cDNA as described previously [20].

### 2.5. Quantitative Real-Time Polymerase Chain Reaction (qPCR)

Expression levels of TNF*α*, IL-1*β*, IL-10, and 18S rRNA were determined using qPCR. Per reaction, 16 ng cDNA was mixed with 5 *μ*l TaqMan Universal Master Mix (Thermo Fisher Scientific), 0.5 *μ*l TaqMan Gene Expression Assay, and 2.3 *μ*l nuclease-free water to a final volume of 10 *μ*l in a 384-well optical reaction plate. Each sample was measured in triplicate wells and underwent 40 cycles of amplification on a ViiA7 real-time PCR system (Thermo Fisher Scientific). Ct values were determined with SDS Software 2.2 (Thermo Fisher Scientific). Target gene expression was normalized to an internal control and 18S RNA as noninducible housekeeping gene.

### 2.6. Protein Expression (Enzyme-Linked Immunosorbent Assay)

TNF*α*, IL-1*β*, IL-6, and IL-10 levels in cell culture supernatants were quantified using commercially available ELISA kits (BD Biosciences, Franklin Lakes, NJ, USA) according to the manufacturer's protocol. Absorption was measured on a Cytation 3 multimode reader.

### 2.7. Oxygen Rate

10^5^ THP-1 cells harvested from passage 3 ((P3), 7 days after initial LPS treatment) were reconstituted in fresh media and transferred to Transwell inserts with 0.4 *μ*m pore size (Corning, NY, USA). Real-time O_2_ measurements were performed using OxoDish® OD24 plates on an SDR reader (PreSens, Heidelberg, Germany) [21]. Plates with cells were equilibrated for 2 h in the incubator (37°C, 5% CO_2_) before stimulation with PBS or LPS (1 *μ*g/ml media). Afterwards, oxygen concentration in the media was measured.

### 2.8. MTT Assay

Reduction of (3-(4,5-dimethylthiazol-2-yl)-2,5-diphenyl tetrazolium bromide (MTT, Sigma) to purple formazan was detected in a colorimetric assay. 100 *μ*l MTT reagent (500 *μ*g MTT/100 *μ*l PBS) was added to 10^5^ cells taken from P3 (96-well plate). After a 2 h incubation period, 100 *μ*l lysis reagent (100 ml N,N-dimethylformamide, 20 g SDS, 2.5 ml 80% acetic acid, and 2.5 ml 1 N HCl in 100 ml ddH_2_O) was supplemented. Plates were analyzed on a microtiter plate reader (BioTek Instruments).

### 2.9. ATP, Lactate, NAD/NADH, and LDH Measurements

ATP content was determined using CellTiter-Glo® Luminescent Cell Viability Assay (Promega, Madison, WI, USA). Lactate-Glo™ Assay (Promega) quantified extracellular lactate. NAD/NADH levels were measured with NAD/NADH-Glo™ Assay (Promega). Lactate dehydrogenase (LDH) was detected with CytoTox96® NonRadioactive Cytotoxicity Assay (Promega). Relative luminescent units (RLU) or absorption was detected on a BioTek Cytation 3 multimode reader. RLU of NAD+ and NADH were normalized to levels measured in unstimulated THP-1 and are presented as arbitrary units (AU). All THP-1 cells utilized in this approach were harvested from P3.

### 2.10. Data Analysis and Statistical Evaluation

Infinium Methylation EPIC Bead Chip Array data were analyzed with GenomeStudio Methylation Module v1.8 and R bioconductor package “minfi” [22]. Differences in cytokine, NAD/NADH, ATP, lactate, LDH, MTT, and oxygen assays were analyzed using one-way analysis of variance with Tukey's multiple comparison test for *n* > 4 or nonparametric Kruskal-Wallis with Dunn's multiple comparisons test for *n* ≤ 4 (Prism 6.0c, GraphPad Software, La Jolla, CA, USA).

### 2.11. Ethical Approval

The study was approved by the local ethic board of the University Hospital Bonn (376/16). All donors provided informed consent.

## 3. Results

### 3.1. LPS Induces Innate Immunotolerance in a Human PBMC In Vitro Model

The first objective of our study was to prove the suitability of our in vitro PBMC model for the induction of long-term LPS tolerance. We aimed to unravel the link between the sustained dysregulated innate immune response as observed in septic patients and the modulation of CpG methylation.

**Figure 2:**
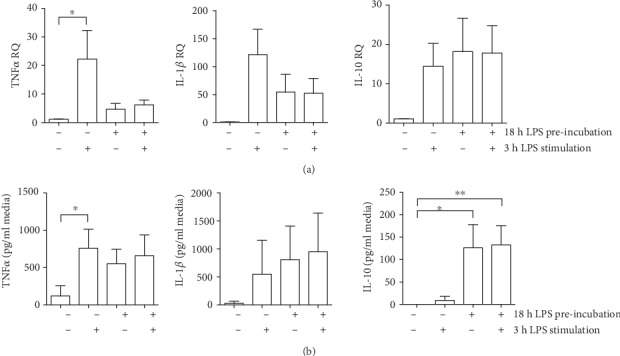
Induction of innate immunotolerance in human PBMCs. PBMCs (10^6^ cells/ml) were isolated from whole blood (*n* = 4 individual donors) and stimulated for 18 h with 10 ng LPS/ml or PBS. Cytokine transcription (a) and secretion (b) were detected upon additional 3 h exposure to LPS or PBS (100 ng/ml). Mean ± SD; *n* = 4; ^∗^*p* < 0.05; ^∗∗^*p* < 0.01.

To prove that 18 h of primary LPS stimulation is suitable for obtaining PBMCs with an LPS-tolerant phenotype, *in vitro* cytokine levels were determined in LPS-tolerized PBMCs vs. control PBMCs (see Figure 1). PBMCs responded to LPS with tolerance induction as reflected by quantification of TNF*α* gene and protein levels. Exposure to a second LPS stimulation did not further elevate TNF*α* transcription (Figure 2(a)) and cytokine secretion (Figure 2(b)) compared to cells treated with primary LPS stimulation alone (*p* < 0.05). IL-1*β* mRNA values demonstrated a similar pattern as TNF*α* with a low responsiveness of LPS-pretreated PBMCs towards a second LPS exposure (not significant). This observation was not reflected by IL-1*β* secretion. 18 h of initial LPS treatment elevated IL-10 gene expression and protein secretion. Restimulation with LPS for 3 h brought IL-10 mRNA transcription to a comparable level.

### 3.2. LPS Preincubation Causes Differential Methylation of Metabolic Pathway-Associated CpG Sites

18 h of LPS incubation caused long-lasting LPS tolerance in PBMCs as proven by cytokine expression assays. Therefore, this point of time was also chosen for CpG methylation profiling. PBMCs donated by healthy volunteers were split, stimulated with either LPS or PBS for 18 h, and harvested for array analysis.

**Figure 3:**
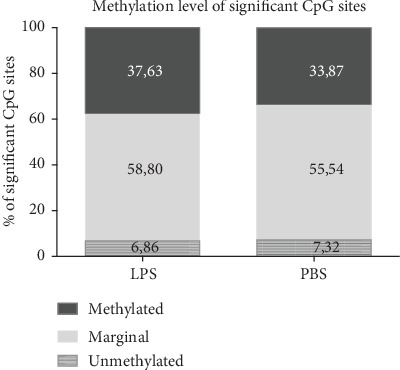
Methylation level of significant CpG sites. Methylome analysis of DNA was performed on PBMCs derived from healthy human donors (*n* = 4).

Processing the array data with Minfi Bioconductor package included quality assessment and correction for age, sex, and single-nucleotide polymorphisms. Afterwards, 1033 CpG sites demonstrated a significant LPS-induced reduction of methylation whereas 1972 CpG sites exhibited significantly enhanced methylation after LPS pretreatment (diff. score > +13 or <-13). Among the significantly modulated CpG sites (cutoff hypomethylated or hypermethylated sites: <-2.0 and >+2.0) in the LPS-pretreated group, 37.63% of the alterations occurred in methylated CpG sites, 6.86% in unmethylated CpG sites, and 58.8% in marginally methylated CpG sites (Figure 3).

Differentially methylated CpG sites corresponding to pathophysiologically and physiologically relevant pathways were analyzed utilizing KEGG mapping. An accumulation of differentially methylated sites in metabolic pathway-related genes emerged (Table 1). Out of 1252 associated sites, 870 were differentially expressed. An enrichment of methylation changes in specific and uniform genomic regions in metabolism-correlated genes did not occur.

### 3.3. Sustained Immunoparalysis in LPS-Tolerant THP-1 Cells

Our array results highlight a correlation of functionally compromised immune cells and differentially methylated CpG sites in metabolic pathways. However, to exclude heterogeneity and evaluate functional relevance of our observations, we continued our studies in a single immortalized monocytic cell line.

**Figure 4:**
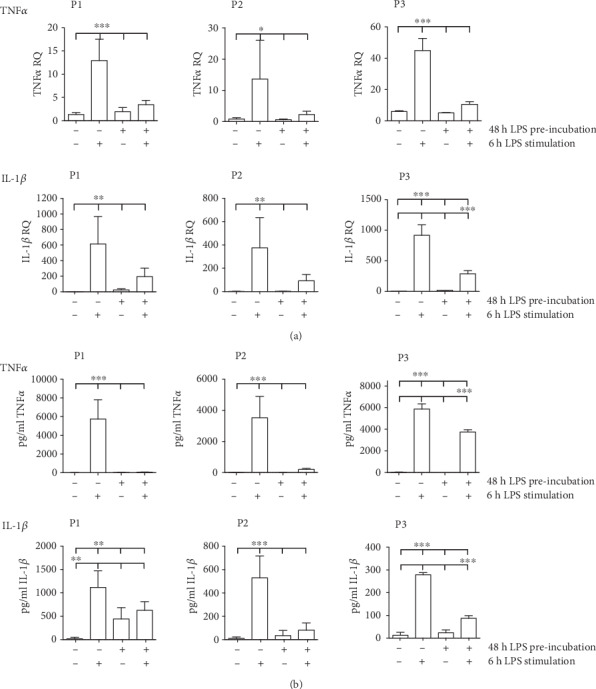
LPS-tolerized (1 *μ*g/ml) THP-1 monocytes (10^6^ cells/ml) demonstrate sustained cytokine mRNA and protein alterations upon 6 h LPS restimulation (1 *μ*g/ml) compared to previously untreated cells. mRNA transcription (a) and protein secretion (b) of TNF*α* and IL-1*β* were measured at different time points after 48 h preincubation followed by resting durations of 0 d (≙ passage (P)1), 2 d (≙ P2), and 5 d (≙ P3) prior to repeated LPS exposure. Mean ± SD; *n* = 6.

First, we established a THP-1 *in vitro* model to induce long-term LPS tolerance. We investigated the sustainment of LPS tolerance up to three passaging cycles after initial endotoxin challenge. Our model takes into account that the majority of cells in P2 and P3 are newly proliferated daughter cells, which possibly carry on LPS tolerance via epigenetic transmission (Supplement Figure 1). Compared to naïve THP-1 cells, restimulated immunoparalysed THP-1 cells responded with a significantly attenuated IL-1*β*and TNF*α* mRNA (Figure 4(a)) and protein expression (Figure 4(b)) upon second LPS treatment in P1, P2, and P3. IL-6 and IL-10 cytokine mRNA levels were rather increased upon another endotoxin challenge in passage 1 (P1) (not significant, Supplement Figure 2). Contrariwise, no considerable alteration of IL-6 and IL-10 was observed between treatment groups in passage 2 (P2). The kinetic of proinflammatory IL-1*β* and TNF*α* expression confirms long-term LPS tolerance in our *in vitro* model.

### 3.4. Alterations of Cellular Metabolism in Endotoxin Tolerance

**Figure 5:**
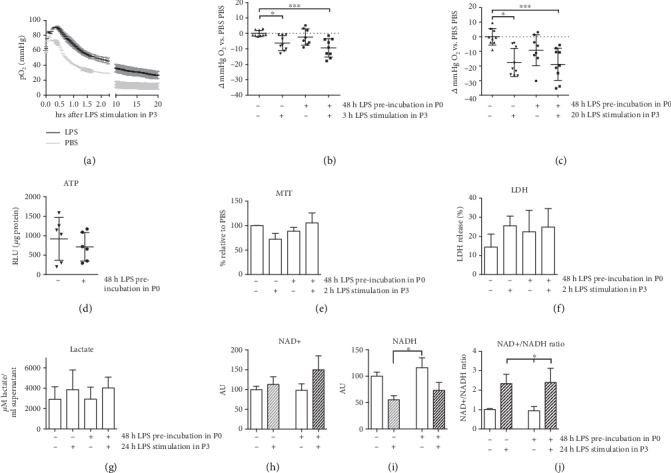
Immunotolerant THP-1 monocytes demonstrate altered metabolism after LPS pretreatment. Cells were stimulated with LPS (1 *μ*g/ml) for 48 h and rested for 5 d after LPS removal. Afterwards, they were challenged with LPS (1 *μ*g/ml) or PBS as indicated. (a) demonstrates a representative measurement of LPS-tolerized and nontolerized cells over 20 h. Oxygen rates (b, c) of monocytes left untreated or preincubated with LPS. Pooled data from (*n* = 9) independent measurements each with 4 technical replicates. MTT assay (b, *n* = 3-5), LDH activity (c, *n* = 4), lactate secretion (d, *n* = 7), and NAD+/NADH (e, *n* = 8) levels were detected. Mean ± SD; ^∗^*p* < 0.05, ^∗∗^*p* < 0.01, and ^∗∗∗^*p* < 0.001.

After demonstrating an ongoing immune dysfunction in monocytes resting for 5 days after 48 h LPS challenge, the effects on cellular metabolism were assessed in cells of P3. First, oxygen consumption as a measurement of cellular respiration was quantified. Cells were stimulated with LPS or PBS at 0 h time point, and O_2_ consumption was continuously measured. An exemplary measurement of nontolerized THP-1 cells stimulated with PBS or LPS is given in Figure 5(a).

Comparing all four treatment groups, maximum oxygen consumption was observed in nontolerized THP-1 cells without additional LPS stimulation. Cells exposed to fresh LPS significantly reduced oxygen consumption compared to naïve cells, both 3 and 20 h after LPS stimulation (Figures 5(b) and 5(c)). LPS-pretreated cells taken from P3 without further LPS stimulation demonstrated intermediate O_2_ uptake not reaching the level of significance after 20 h of continuous measurement (Figures 5(b) and 5(c)). However, tolerized and nontolerized cells taken from P3 responded with a similar oxygen consumption towards a second LPS stimulus at 0 h.

We assumed that this observation came along with quantitative changes in the synthesis of ATP and NADH or enzymes participating in energy metabolism. However, ATP levels quantified in endotoxin pretreated and naïve THP-1 cells taken from P3 revealed no major differences between treatment groups (*n* = 6; Figure 5(d)).

MTT tests reflect the metabolic activity in cells, mainly dependent on NAD(P)H reduction equivalents and oxidoreductase activity. Results in treatment groups were expressed relative to values obtained from the PBS pretreated group. No difference between LPS-pretreated and naïve cells was observed (Figure 5(e)). Two hours of LPS stimulation slightly attenuated the rate of reduced MTT in naïve cells (not significant) and was unchanged in LPS-pretreated THP-1 cells from P3.

Intracellular LDH uses lactate and NAD+ to synthetize pyruvate and NADH. Upon cytotoxic stimuli, LDH is released from cells. In our model, LDH remained slightly increased (1.5-fold, *n* = 4, not significant) in LPS-pretreated THP-1 cells compared to PBS-pretreated cells (Figure 5(f)). Two hours of additional LPS treatment 7 d after pretreatment affected LDH activity in the same moderate way.

Lactate is an end product of glycolysis with regulatory properties of glucose homeostasis and energy production. To check for lactate, NAD+, and NADH accumulation in the media, we performed restimulation with LPS of tolerant and nontolerant THP-1 cells for 24 h. No differences in lactate concentration in the media were detected 7 d after prestimulation, and 24 h of additional LPS stimulation did not cause any relevant increase of lactate level either (Figure 5(g)).

In line with this, intracellular NAD+ and NADH rates were not altered in tolerant and nontolerant THP-1 cells (Figures 5(h)–5(j)). LPS restimulation of both, tolerized and non-tolerized cells, significantly elevated the NAD+/NADH ratio, i.e., the reduction of NAD^+^ to NADH was decreased overall (Figure 5(j)).

Despite ongoing immune dysfunction in our model of LPS-tolerized THP-1 cells, the chosen readouts for cellular metabolism did not reveal any noteworthy alterations between tolerant and nontolerant THP-1 monocytes.

## 4. Discussion

Monocytes belong to the first line of defense against invading pathogens, by orchestrating the inflammation and contributing to sepsis outcome [23]. Monocytes from septic patients can exhibit endotoxin tolerance, compromising their function and paralyzing the immune response. Endotoxin tolerance and its influence on sepsis have already been studied [24]. However, the correlation of immunoparalysis, metabolic dysregulation, and epigenetic modifications in monocytes isolated from septic patients is a matter of ongoing investigation.

LPS can reprogram the inflammatory response toward a reduced inflammatory cytokine production (decreased TNF*α* production) in response to subsequent LPS challenge [25]. In our model of isolated human PBMCs, we detected attenuated TNF*α* and IL-1*β* cytokine mRNA expression and TNF*α* secretion upon LPS restimulation of tolerized PBMCs. The proinflammatory cytokines TNF*α* and IL-1*β* are accepted markers for demonstrating endotoxin tolerance [26, 27], while IL-10 reflects the anti-inflammatory cell response. Due to the specific properties of IL-1*β* processing and activation through the inflammasome [28], reduction of IL-1*β* protein as a result of attenuated mRNA transcription occurs temporally delayed.

The arising question was whether and how epigenetic mechanisms play a role in these observations. Epigenetic reprogramming may establish a form of innate immune memory against systemic infection or inflammation [29]. Histone and DNA methylation interact to silence TNF*α* expression [30]. Our results are in line with the observation that monocyte capacity to release anti-inflammatory mediators (e.g., IL-10) is neither impaired nor enhanced after sepsis and support the assumption that monocyte intracellular signaling is shifted from inflammatory response toward the production of anti-inflammatory molecules as a characteristic of tolerization [6, 31].

In order to link LPS tolerance with epigenetic modifications in PBMCs, we performed a CpG methylation array. DNA methylation is a stable epigenetic mark that is inheritable to daughter cells and changes DNA accessibility, thereby driving promoter activity. So far, methylation changes in human PBMCs manifested 18 h upon LPS challenge have not been examined. Our methylation array revealed that differential methylation patterns occurred in metabolic pathway-associated genes in LPS-tolerized PBMCs. Metabolic pathway reprogramming and metabolic modulation of immune cell function have been of accelerating interest in the past few years [17, 32]. Defects in leukocyte energy metabolism are closely linked to the initial activation of the host defense and contribute to immunoparalysis in septic patients [15]. Due to interindividual variability in response to LPS stimulation as well as a low sample number, single CpG site methylation modifications did not reach significant values. This observation is reflected by Grondman et al., who also documented a heterogenic metabolic response in monocytes from healthy subjects rendered immunotolerant [33]. Tissue, age, and sex-specific DNA methylation changes have been reported. Nonetheless, KEGG pathway analysis revealed the highest differential methylation rate in CpG sites related to metabolic pathways.

Since DNA methylation is a stable epigenetic mark and can be passed on through multiple cell divisions, we suspected that LPS-induced alterations of metabolic activity may persist. Therefore, we aimed to develop a cell culture model that allows for long-term observations of LPS tolerance phenomena including metabolic activity in monocytic cells. Since vitality of PBMCs is known to rapidly decrease after 36 h, we established a model of extended LPS tolerance with repeatedly passaged monocytic THP-1 cells. First, we characterized the reliability of our model by detecting the standard parameters of endotoxin tolerance, observing that TNF*α* and IL-1*β* cytokine repression upon LPS restimulation was still measurable 7 days after initial LPS stimulation. Assuming a doubling of THP-1 cells within approximately 2 days, the observed variation in TNF*α* and IL-1*β* cytokine levels was not limited to parent cells with immediate contact to the stimulant but was also monitored in newly proliferated daughter cells.

In addition to the widely accepted response of monocytes to TLR4 stimulation by proinflammatory cytokine release, cells activated by LPS also undergo profound metabolic changes [34]. Activation of macrophages and dendritic cells by proinflammatory stimuli causes them to undergo a metabolic switch towards glycolysis [35]. Besides mere energy and biosynthetic precursor generation, there is growing evidence that metabolites also control immunological effector function [36]. With regard to our cytokine expression kinetics and based on our methylation array findings, we hypothesized that THP-1 cells tolerized with LPS demonstrate with a profound metabolic switch after 7 d.

Presumably, the significantly decreased O_2_ uptake of tolerized cells after LPS restimulation points towards an ongoing inflammatory phenotype with a reduced rate of oxidative phosphorylation and coverage of energy demand via glycolysis. However, our data highlight that in response to LPS pre-treatment key glycolytic enzymes, cofactors and molecules involved in energy transfer were not altered after 7 d, whereas LPS restimulation caused a reduction of NAD+ to NADH conversion. These observations are not congruent with our hypothesis, arising new questions. We are aware that assay results strongly depend on the time point of analysis chosen. As demonstrated by Zhu et al., metabolism and bioenergetics of monocytes sequentially activate, deactivate, and ultimately resolve acute inflammation [34]. One additional aspect of interest, which has to be examined in future projects, is the host iron metabolism. By deregulating glucose metabolism, this mechanism has been shown to compromise the establishment of disease tolerance to sepsis [37, 38].

In vitro models often lack clinical relevance due to various methodological limitations. Most studies analyze short time intervals after LPS stimuli, which is barely avoidable when experimenting with PBMCs. On the other hand, immortalized cell lines do not share all physiologic properties of primary cells. THP-1 cells, which serve as a model for human monocytes, derived from a spontaneously immortalized monocyte-like cell line, developed in a case of acute monocytic leukemia [18]. They respond weakly to LPS due to low CD14 expression levels [39], which entails different LPS dosages to correct for the differences in LPS responsiveness. Thus, we increased the widely used LPS dosage of 10 ng/ml in PBMC experiments to 1 *μ*g/ml in THP-1 experiments [40]. Furthermore, comparisons of THP-1 cells to freshly isolated PBMCs and monocytes revealed that these cells may release different cytokine quantities under identical experimental conditions [41]. Thus, clinical implications may better be inferred from cumulated experiments performed with primary, immortalized, and septic patient cell samples.

## 5. Conclusions

Our model reveals that LPS tolerance effects reflected on the level of cytokine regulation outlast metabolic alterations. Immune response and metabolic regulation are highly integrated and tend to reestablish homeostasis. Hence, the selected THP-1 model is appropriate when examining the termination of inflammatory processes in a single cell model under defined experimental conditions. Snapshot collections as well as large-scale array analysis of intertwined immunological reactions, epigenetic modifications, metabolic changes, and kinetics will steadily give further insight into sepsis pathophysiology.

## Figures and Tables

**Table 1:** Upon 18 h LPS tolerization (10 ng LPS/ml media or PBS as control, 106 cells/ml), methylome analysis of DNA derived from healthy human PBMC donors (*n* = 4) was performed and KEGG pathway results are presented.

		Top KEGG pathways	*N*	DE	P.DE	FDR
1	path:hsa01100	Metabolic pathways	1252	870	6,27*E*-255	2,01*E*-252
2	path:hsa05200	Pathways in cancer	394	316	1,20*E*-97	1,93*E*-95
3	path:hsa05165	Human papillomavirus infection	318	244	1,79*E*-66	1,91*E*-64
4	path:hsa04151	PI3K-Akt signaling pathway	339	243	9,24*E*-59	7,39*E*-57
5	path:hsa04010	MAPK signaling pathway	255	197	5,27*E*-50	3,37*E*-48
6	path:hsa05166	HTLV-I infection	252	192	1,05*E*-49	5,63*E*-47
7	path:hsa04360	Axon guidance	174	151	2,22*E*-45	1,01*E*-43
8	path:hsa04144	Endocytosis	244	186	4,18*E*-45	1,67*E*-43
9	path:hsa04015	Rap1 signaling pathway	210	167	1,86*E*-44	6,61*E*-42
10	path:hsa04014	Ras signaling pathway	226	173	2,24*E*-43	7,15*E*-42
11	path:hsa05169	Epstein-Barr virus infection	197	156	3,43*E*-41	9,97*E*-42
12	path:hsa04510	Focal adhesion	198	159	4,35*E*-41	1,16*E*-38
13	path:hsa05205	Proteoglycans in cancer	203	157	1,11*E*-37	2,74*E*-36
14	path:hsa04810	Regulation of actin cytoskeleton	211	160	8,19*E*-37	1,87*E*-35
15	path:hsa05202	Transcriptional misregulation in cancer	181	141	7,12*E*-36	1,52*E*-34
16	path:hsa05203	Viral carcinogenesis	198	149	1,27*E*-35	2,53*E*-34
17	path:hsa05167	Kaposi's sarcoma-associated herpesvirus infection	186	140	2,96*E*-35	5,58*E*-34
18	path:hsa04022	cGMP-PKG signaling pathway	162	132	1,17*E*-35	2,09*E*-34
19	path:hsa05016	Huntington's disease	186	140	1,65*E*-34	2,79*E*-33
20	path:hsa04141	Protein processing in endoplasmic reticulum	165	131	1,85*E*-34	2,96*E*-33

KEGG pathway: code from Kyoto Encyclopedia of Genes and Genomes; *N*: number of KEGG pathway-related CpG sites contained in array; DE: number of differentially methylated CpG sites; P.DE: adjusted differential methylation *p* value; FDR: false discovery rate.

## Data Availability

The data used to support the findings of this study are available from the corresponding author upon request.

## Supplementary Materials

Supplementary Materials Supplement Figure 1: THP-1 proliferation recovers within 5 days after 48 h of LPS stimulation. LPS or PBS were added to media in P0 for 48 h. Cell density was determined in all treatment groups at the end of P0 (48 h after treatment begin), P1 (96 h after treatment begin), and P2 (144 h after treatment begin) prior to resuspension in fresh media containing no LPS at a density of 106 cells/ml (A) (mean ± SEM, *n* = 8-12; ^∗^*p* < 0.05). At each time point, statistical differences between PBS and LPS treated cells were calculated with a *t*-test (^∗^*p* < 0.05). Supplement Figure 2: LPS-tolerized (1 *μ*g/ml) THP-1 monocytes demonstrate sustained IL-6 and IL-10 cytokine mRNA and protein alterations upon LPS restimulation (1 *μ*g/ml) compared to previously untreated cells. mRNA transcription of IL-6 and IL-10 was measured in passage (P)1 and P2 after 48 h preincubation followed by resting durations of 0 h (P1) and 48 h (P2). Mean ± SD; *n* = 3.
