# Supplementary material for: Sustained Immunoparalysis in Endotoxin-Tolerized Monocytic Cells
Source: Mediators Inflamm. 2020 Jun 13;2020:8294342. doi: 10.1155/2020/8294342 (PMC7306843; doi:10.1155/2020/8294342)
Supplement: Supplementary Materials — Supplement Figure 1: THP-1 proliferation recovers within 5 days after 48 h of LPS stimulation. LPS or PBS were added to media in P0 for 48 h. Cell density was determined in all treatment groups at the end of P0 (48 h after treatment begin), P1 (96 h after treatment begin), and P2 (144 h after treatment begin) prior to resuspension in fresh media containing no LPS at a density of 106 cells/ml (A) (mean ± SEM, n = 8-12; ∗p < 0.05). At each time point, statistical differences between PBS and LPS treated cells were calculated with a t-test (∗p < 0.05). Supplement Figure 2: LPS-tolerized (1 μg/ml) THP-1 monocytes demonstrate sustained IL-6 and IL-10 cytokine mRNA and protein alterations upon LPS restimulation (1 μg/ml) compared to previously untreated cells. mRNA transcription of IL-6 and IL-10 was measured in passage (P)1 and P2 after 48 h preincubation followed by resting durations of 0 h (P1) and 48 h (P2). Mean ± SD; n = 3. [file 8294342.f1.pdf]

Supplementary figures

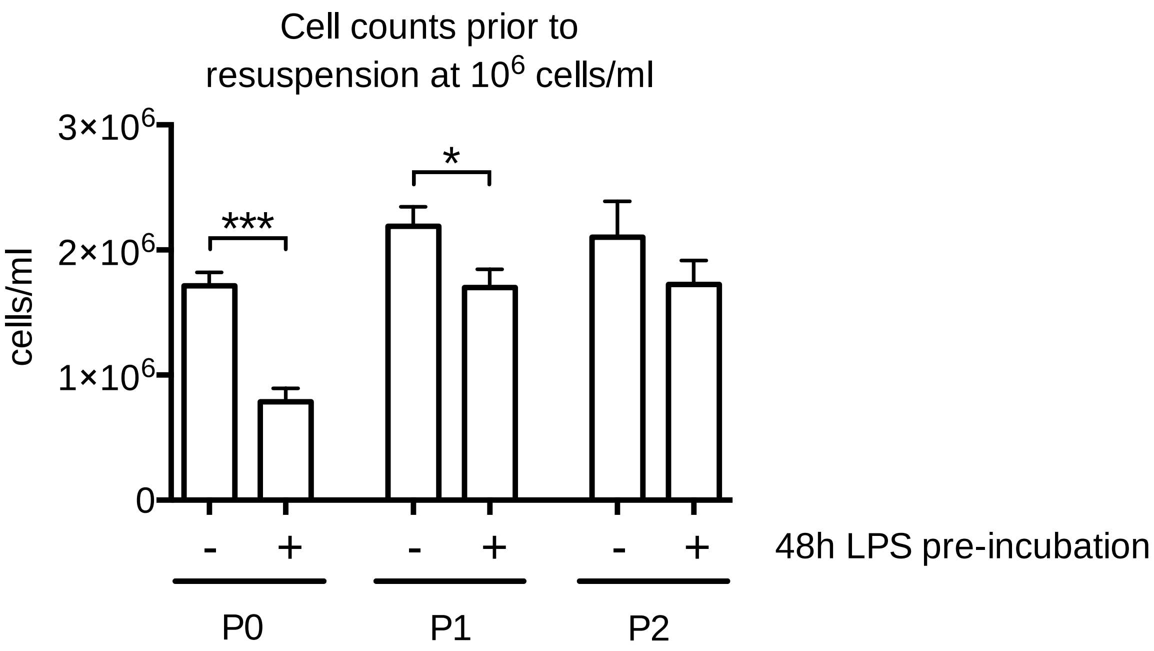

Suppl. Figure 1. THP-1 proliferation recovers within 5 days after 48 h of LPS stimulation. LPS or PBS were added to media in P0 for 48 h. Cell density was determined in all treatment groups at the end of P0 (48 h after treatment begin), P1 (96 h after treatment begin) and P2 (144 h after treatment begin) prior to resuspension in fresh media containing no LPS at a density of 10^6 cells/ml (A)(Mean ± SEM, n = 8-12; * $p < 0.05$). At each time point statistical differences between PBS and LPS treated cells were calculated with t-test (* $p < 0.05$).

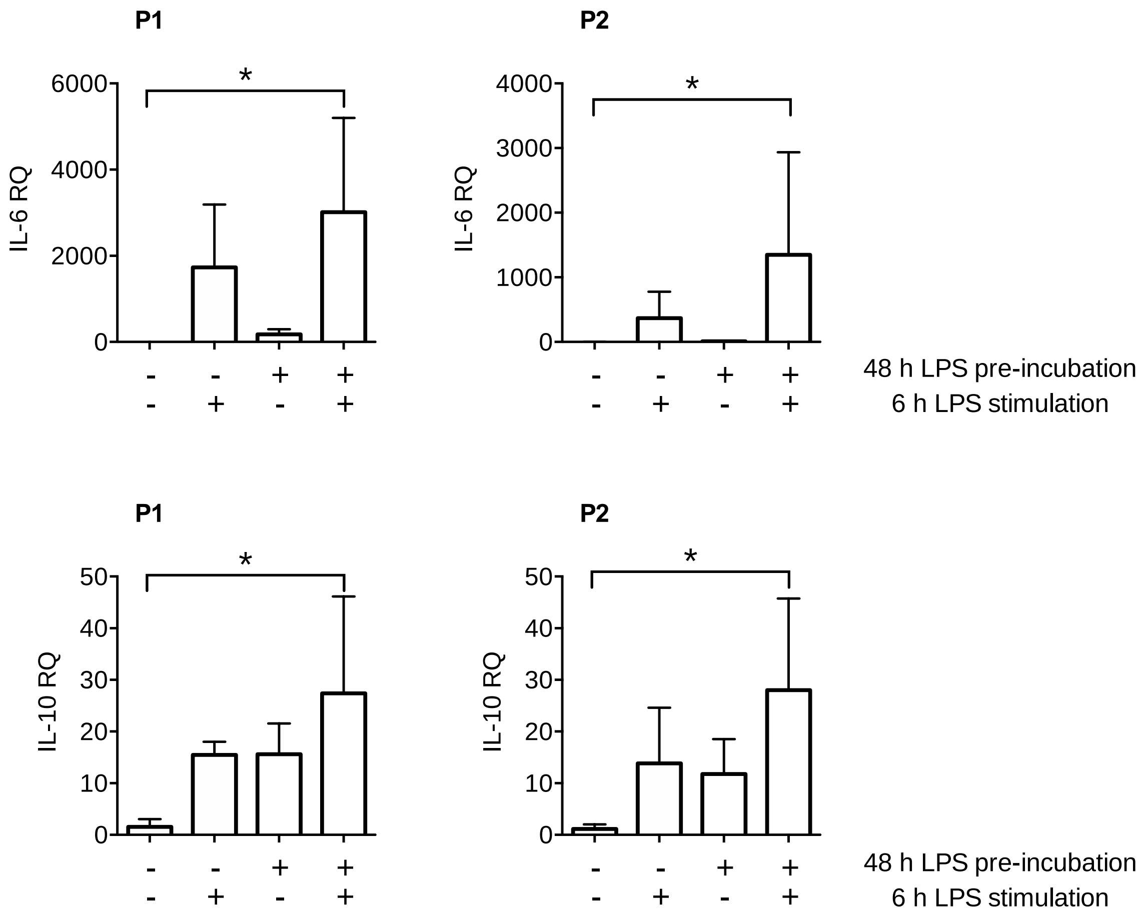

Suppl. Figure 2. LPS tolerized (1 µg/ml) THP-1 monocytes demonstrate sustained IL-6 and IL-10 cytokine mRNA and protein alterations upon LPS restimulation (1 µg/ml) compared to previously untreated cells. mRNA-transcription of IL-6 and IL-10 was measured in passage (P) 1 and 2 after 48 h preincubation followed by resting durations of 0 h (P1) and 48 h (P2). Mean ± SD; n = 3.
